# Antibodies against citrullinated proteins in relation to periodontitis with or without rheumatoid arthritis: a cross-sectional study

**DOI:** 10.1186/s12903-021-01712-y

**Published:** 2021-07-20

**Authors:** Pit Hui Lew, Mohammad Tariqur Rahman, Syarida Hasnur Safii, Nor Adinar Baharuddin, Peter Mark Bartold, Sargunan Sockalingam, Noor Lide Abu Kassim, Rathna Devi Vaithilingam

**Affiliations:** 1 Department of Restorative Dentistry, Faculty of Dentistry, University of Malaya, 50603 Kuala Lumpur, Malaysia; 2 Dean’s Office, Faculty of Dentistry, University of Malaya, 50603 Kuala Lumpur, Malaysia; 3 Department of Dentistry, University of Adelaide, Adelaide, Australia; 4 Department of Rheumatology, Faculty of Medicine, University of Malaya, 50603 Kuala Lumpur, Malaysia; 5 Kulliyyah of Education, International Islamic University Malaysia, 53100 Kuala Lumpur, Malaysia

**Keywords:** Anti-citrullinated protein antibodies (ACPA), Citrullination, Periodontitis, Rheumatoid arthritis

## Abstract

**Background:**

Previous studies have reported conflicting findings between serum anti-citrullinated protein antibodies (ACPA) levels in rheumatoid arthritis (RA) participants with and without periodontitis (Pd). This study aimed to analyse possible correlations between serum ACPA levels and clinical parameters in Pd and RA participants.

**Methods:**

Full mouth periodontal examination (probing pocket depth, clinical attachment levels, gingival bleeding index, visual plaque index) was conducted and serum samples obtained from 80 participants comprising RA, Pd, both RA and Pd (RAPd) and healthy individuals (HC). Erythrocyte sedimentation rates (ESR) and periodontal inflamed surface area (PISA) were obtained. Serum samples were analysed for ACPA quantification using enzyme-linked immunosorbent assay (ELISA).

**Results:**

Median levels (IU/mL) of ACPA (interquartile range, IQR) in RAPd, RA, Pd and HC groups were 118.58(274.51), 102.02(252.89), 78.48(132.6) and 51.67(91.31) respectively. ACPA levels were significantly higher in RAPd and RA as compared to HC group (*p* < 0.05). However, ACPA levels of any of the groups were not correlated with any clinical periodontal and RA parameters within the respective groups.

**Conclusions:**

At individual level, the amount of serum ACPA seem to have an increasing trend with the diseased condition in the order of RAPd > RA > Pd > HC. However, lack of any significant correlation between the serum ACPA levels with the clinical Pd and RA parameters warrants further studies to investigate the causal link between RA and Pd for such a trend. Further studies involving more inflammatory biomarkers might be useful to establish the causal link between Pd in the development and progression of RA or vice versa.

**Supplementary Information:**

The online version contains supplementary material available at 10.1186/s12903-021-01712-y.

## Background

Periodontitis (Pd) is a host-mediated chronic inflammatory disease associated with a dysbiotic dental biofilm and is characterised by both soft and hard tissue destruction around the teeth [1]. Current epidemiological evidence suggests that Pd is moderately prevalent globally while its severe subtype displays a prevalence of 11.2% [2]. Rheumatoid arthritis (RA) is a chronic inflammatory autoimmune disease which is characterised by inflamed synovial tissues as well as destruction of cartilage and bone in the joints. It has a global prevalence of 1% and may lead to severe disabilities and early premature mortality as it progresses with age [3].

Numerous epidemiological and clinical studies have reported a significant association between Pd and RA whereby Pd is more common and severe in patients with established RA and vice versa [4, 5]. Despite the differences in their initiating aetiological mechanisms, both chronic inflammatory conditions share a similar host mediated pathogenesis characterised by similar sets of pro-inflammatory cytokines, and risk factors (such as smoking, obesity and ageing), that justify a plausible link between them [6] which subsequently also impacts their oral health related quality of life [7, 8]. However, the actual mechanisms through which RA and Pd are interrelated is still unclear.

Bright and colleagues [9] have proposed the dual hit hypothesis for pathogenesis of RA being driven by protein citrullination or carbamylation, or both, in inflamed periodontal tissues. Upon citrullination, a post translational modification of amino acid arginine to citrulline mediated by peptidyl arginine deiminase enzymes (PADs), citrullinated proteins evoke autoimmune response in a susceptible patient. As a result, pathogenic T and B cells are activated, leading to the formation of RA-specific anti-citrullinated protein antibodies (ACPA) [10]. Detection of these antibodies in the serum of participants with RA has been well documented [11, 12]. Interestingly, the presence of these same autoantibodies in the serum of participants with periodontitis has also been reported in several studies [13–15].

In view of the possible causal link between RA and Pd via citrullination, various studies have been carried out to assess and compare the ACPA levels in RA participants with and without Pd. In a case–control study conducted among a cohort of non-smoker RA participants, ACPA levels were reported to be significantly higher in RA participants with Pd than those without Pd, suggesting that Pd and high ACPA expression could serve as a potential environmental trigger in RA development [16]. However, two other studies conducted to investigate the levels of ACPA in RA participants with and without Pd did not find significantly higher levels of ACPA in RA-Pd participants as compared to RA non-Pd participants [17, 18]. As a result of these conflicting findings, this study was conducted to evaluate and compare the levels of serum ACPA in four groups of participants (RAPd, RA, Pd and HC groups) as well as to identify the possible association between RA and Pd by correlating the serum ACPA levels to both clinical RA and Pd parameters.

## Methods

### Study design

This was a cross-sectional comparative study conducted at Faculty of Dentistry, University of Malaya, Malaysia. Study was conducted in accordance with the Declaration of Helsinki. Ethics approval was obtained from the Medical Research Ethics Committee (MREC), University Malaya Medical Centre (UMMC) [MRECID NO. 2017510–5227) and Medical Ethics Committee, University of Malaya’s Faculty of Dentistry [DF-RD1707/0029(L)]. Participants were recruited between November 2017 and December 2018 from Primary Care Unit, Faculty of Dentistry, University of Malaya (Pd and HC groups) and Rheumatology Clinic, University Malaya Medical Centre (RA and RAPd groups).

### Sample size calculation

Sample size calculation was performed based on the mean levels and standard deviations (SD) of serum ACPA in RA group (86.0 ± 73.0 U/mL) and non-RA group (7.5 ± 7.4 U/mL) reported by Karkucak [19]. The required number of samples stands at 20 for each group in order to provide 80% power at a significance level of 5%.

### Participants recruitment

**Fig. 1:**
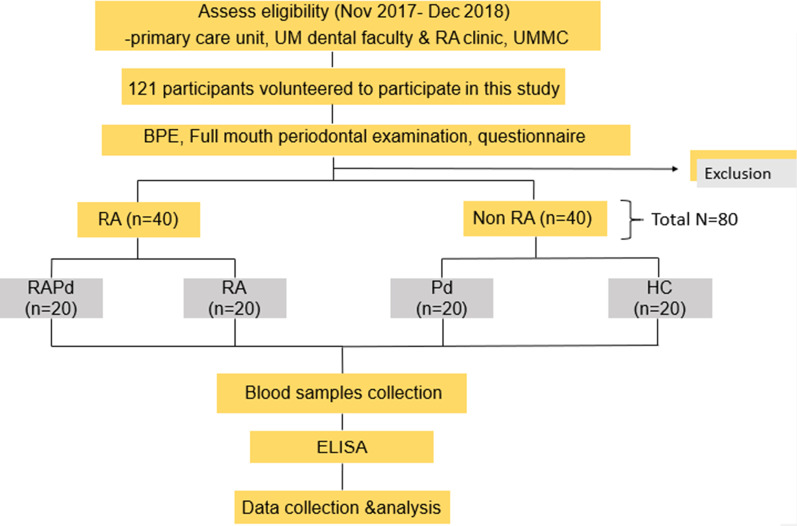
Study flow chart showing the summary of the study design. RA, rheumatoid arthritis; Pd, periodontitis; RAPd group, subjects with RA and Pd; RA group, subjects with RA but without Pd; Pd group, subjects without RA but has Pd; HC group, subjects without both RA and Pd; UM, University of Malaya; UMMC, University of Malaya Medical Centre; BPE, basic periodontal examination; ELISA, enzyme link immunosorbent assay

A total of 80 participants (20 from each of four groups) who fulfilled the inclusion criteria were enrolled in this study (groups: RAPd group, RA group, Pd group, and HC group). The summary of the recruitment process and study design was demonstrated in flow chart (Fig. 1).

The primary inclusion criteria included: age ≥ 30 years with at least eight teeth excluding third molars. All participants with RA met the 2010 RA Classification Criteria of the American College of Rheumatology/European League Against Rheumatism (ACR/EULAR) [20] with > 1 year of diagnosis. In the Pd group, participants were diagnosed with moderate to severe chronic periodontitis [21] or Stage II to stage IV periodontitis [22]. In the RA and HC groups, participants were either periodontally healthy or had gingivitis with PPD ≤ 3 mm.

Exclusion criteria were pregnancy and lactation, concurrent systemic or debilitating medical conditions such as diabetes mellitus and other autoimmune diseases or malignancies, periodontal treatment within the past four months, and the use of antibiotics within the past four months. Written informed consent was obtained from each participant before the study.

### Standardisation of examiners

Three trained and calibrated examiners including LPH (the first author of the manuscript) were involved in the patient examination and data collection. Kappa values of more than 0.75 was obtained by all three examiners for both intra-examiner and inter-examiner standardizations of PPD and CAL and thus were considered “reproducible” and “standardized”.

### Clinical examination/measurements

All participants were required to complete a questionnaire which consisted of socio-demographic information (age, gender, and ethnicity), medical and dental histories, including lifestyle practices or habits (smoking status and oral hygiene habits). Subsequently, all eligible participants were subjected to a full mouth periodontal examination on 6 sites (mesio-buccal, mid-buccal, disto-buccal, mesio-lingual/palatal, mid-lingual/palatal, disto-lingual/palatal) of each tooth. All teeth were examined except third molars.

Clinical periodontal parameters measured were Visible Plaque Index (VPI) [23], Gingival Bleeding Index (GBI) [23], Probing Pocket Depth (PPD) and Clinical Attachment Level (CAL). UNC 15 colour-coded periodontal probe (Hu-Friedy, Chicago, USA) was used in all the measurements. The amount of inflamed periodontal tissue was quantified as the periodontal inflamed surface area (PISA) whereby the surface area of bleeding pocket epithelium was calculated in square millimeters, illustrating the inflammatory burden posed by periodontitis [24, 25]. For RA participants, erythrocyte sedimentation rate (ESR), C-reactive protein (CRP), and disease duration of the disease were collected from their medical records.

### Enzyme Linked Immunosorbent Assay (ELISA)

Peripheral blood (10 mL) was obtained from venous cubital fossa of each subject. After coagulation, the blood sample was centrifuged for 15 min at 1000 × g. The serum was aliquoted into labelled microcentrifuge tubes (1.5 μL) and stored at − 80 °C until analysis. Commercially available ELISA kits (Human CCP-Ab ELISA Kits; Elabscience, Wuhan, China) were used to analyse serum samples for the presence and quantification of ACPA. ELISA was performed following the manufacturer’s instructions. Briefly, the diluted samples were incubated in the pre-coated ELISA plates. Standards or samples were added to the appropriate ELISA plate wells and combined with the specific antigen. Then, a biotinylated detection antigen specific for ACPA and Avidin-Horseradish Peroxidase (HRP) conjugate were added to each microplate well successively and incubated. After incubation, free components were washed away. Then, the substrate solution was added to each well. The enzyme–substrate reaction is terminated by adding a stop solution. All standards and serum samples were assayed in triplicates. The absorbance was recorded using a microplate reader at 450 nm wavelength. Standard curve was generated using standard optical density (OD). The OD value is proportional to the concentration of ACPA. The concentration of ACPA in serum samples was determined by comparing the OD of the samples to the standard curve. The cut off value for ACPA positivity was 68.73 ± 52.49 IU/mL.

### Statistical analyses

Statistical analysis was performed using SPSS 25.0 statistical software (IBM, Chicago, IL, USA). All the metric demographic and clinical data were checked for normal distribution using the Kolmogorov–Smirnov test. One-way ANOVA was used to compare VPI, GBI, PPD and CAL between the four groups while Kruskal–Wallis test was used for PISA comparison between groups. Since the ESR was normally distributed, independent sample *T* test was used to compare the mean ESR between RAPD and RA groups.

ACPA levels for all participants were plotted as median and interquartile range (IQR) as all metric values were not normally distributed. For statistical evaluation of the pairwise difference, the Pairwise Mann–Whitney *U* test was used to compare the medians of ACPA levels between groups for all possible pairs. The differences in the distributions of categorical outcomes were analysed using the Pearson chi-square test while differences in continuous outcomes between the four groups were determined by using one-way ANOVA test. The correlation between the serum level of ACPA and clinical periodontal and RA parameters were evaluated using Spearman rho correlation test. All tests were two-sided, and results with *p* < 0.05 were considered statistically significant at 95% confidence interval (CI).

## Results

### Characteristics of participants

Participants from the Pd and HC groups were generally younger than RA group. Most participants in all four groups were females while those in the RAPd and Pd groups were diagnosed with localised moderate to severe Pd. Although the minimum number of teeth required in our study was 8 teeth, all the participants involved in this study had at least 13 teeth.

There were statistically significant differences in age and gender between the four groups (*p* < 0.05), however, no statistically significant differences were observed for education level, ethnicity, smoking status, mean ESR, duration of RA and types of RA medication used (Table 1).

**Table 1:** Sample characteristics of study population

Group/Characteristics	RAPd	RA	Pd	HC	*p*-value
	(n = 20)	(n = 20)	(n = 20)	(n = 20)	
Mean age (years)	54.3 ± 7.5	52.7 ± 9.5	44.0 ± 11.6	39.6 ± 11.5	** < 0.00^a^
Mean ESR (mm/hr)	32.8 ± 21.4	27.9 ± 11.6	N/A	N/A	0.379^b^
	n (%)	n (%)	n (%)	n (%)	
Gender					
Female	14 (70)	19 (95)	11 (55)	16 (80)	**0.028^c^
Male	6 (30)	1 (5)	9 (45)	4 (20)	
Ethnicity					
Malay	4 (20)	7 (35)	9 (45)	13 (65)	0.086^c^
Chinese	11 (55)	7 (35)	9 (45)	5 (25)	
Indian	5 (25)	6 (30)	2 (10)	2 (10)	
Educational level					
Tertiary	6 (30)	13 (65)	13 (65)	18 (90)	0.050^c^
Secondary	14 (70)	6 (30)	7 (35)	2 (10)	
Primary	0 (0)	1 (5)	0 (0)	0 (0)	
Smoking status					
Smoker	2 (10)	0 (0)	3 (15)	3 (15)	0.457^c^
Former smoker	2 (10)	0 (0)	1 (5)	1 (5)	
Non-smoker	16 (80)	20 (100)	16 (80)	16 (80)	
RA disease duration					
< 5	8 (40)	7 (35)	N/A	N/A	0.587^c^
5–10	6 (30)	9 (45)	N/A	N/A	
> 10 years	6 (30)	4 (20)	N/A	N/A	
RA medication Corticosteroid NSAIDs Conventional DMARDs Biologic DMARDs Combination	1(5) 3(15) 7(35) 0(0) 6(30)	0(0) 2(10) 9(40) 1(5) 5(25)	N/A N/A N/A N/A N./A	N/A N/A N/A N/A N/A	0.632^c^
PD status					
Localised Pd	17 (85)	N/A	14 (70)	N/A	0.110^c^
Generalised Pd	3 (15)	N/A	6 (30)	N/A	

RA, Rheumatoid arthritis; Pd, Periodontitis; RAPd group, Subjects with RA and Pd; RA group, Subjects with RA but without Pd; Pd group, Subjects without RA but has Pd; HC group, Subjects without both RA and Pd; ESR, Erythrocyte sedimentation rate; NSAIDs, Non-steroidal anti-inflammatory drugs; DMARDs, Disease modifying antirheumatic drugs

^a^One-way ANOVA

^b^Independent sample *T*-test

^c^Pearson Chi-square test

**Statistically significant at *p* < 0.05

### Clinical periodontal parameters

The mean (± SD) PISA was found to be the highest in the Pd group (1148.53 ± 782.47mm^2^) followed by RAPd group and HC group. The RA group was found to have the lowest mean PISA score among the four groups with the mean values of 102.09 ± 96.56mm^2^. Pd group was found to have the highest mean PPD and CAL followed by RAPd group. On the other hand, both the RA and HC groups were found to have similar mean values of PPD and CAL, which were much lower than the periodontitis group as expected. Statistically significant differences (*p* < 0.05) were observed for all the clinical periodontal parameters measured (VPI, GBI, PPD, CAL and PISA) between the four groups of participants (Table 2).

**Table 2:** Comparison of the clinical periodontal parameters between groups

Periodontal parameters	RAPd (n = 20)	RA (n = 20)	Pd (n = 20)	HC (n = 20)	*p-*value
	Means ± SD	Means ± SD	Means ± SD	Means ± SD	
VPI (%)	55.24 ± 25.13	36.04 ± 23.17	55.18 ± 26.94	28.47 ± 20.78	**0.001^a^
GBI (%)	33.52 ± 23.41	7.29 ± 6.54	40.02 ± 24.45	12.92 ± 15.39	** < 0.001^a^
PPD (mm)	3.07 ± 0.72	1.92 ± 0.28	3.62 ± 0.92	1.95 ± 0.32	** < 0.001^a^
CAL (mm)	3.77 ± 1.12	0.61 ± 0.10	4.33 ± 1.78	0.63 ± 0.19	** < 0.001^a^
PISA (mm^2^)	772.83 ± 619.45	102.09 ± 96.56	1148.53 ± 782.47	202.80 ± 137.76	** < 0.001^b^

RA, Rheumatoid arthritis; Pd, Periodontitis; RAPd group, Subjects with RA and Pd; RA group, Subjects with RA but without Pd; PD group, Subjects without RA but has Pd; HC group, Subjects without both RA and Pd; VPI, Visible plaque index; GBI, Gingival bleeding index; PPD, Probing pocket depth; CAL, Clinical attachment level; PISA, Periodontal inflamed surface area

^a^One-way ANOVA

^b^Kruskal- Wallis Test

**Statistically significant at *p* < 0.05

### Serum ACPA levels

**Fig. 2:**
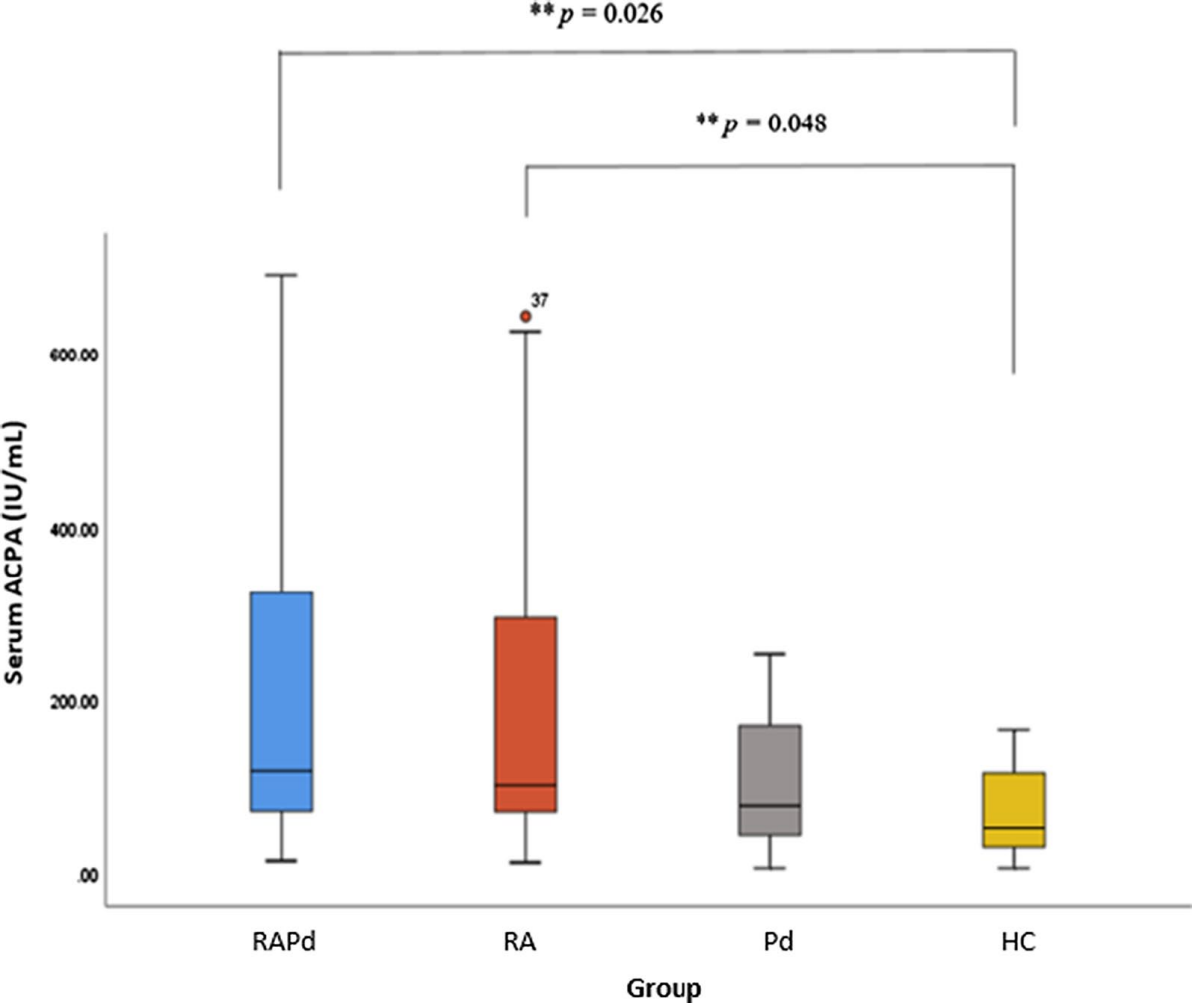
Boxplots of the serum anti-citrullinated protein antibody (ACPA) levels in four groups of subjects. The medians of ACPA levels between the groups were compared with Kruskal–Wallis test (between group, *p* = 0.005), which indicates significant difference between groups at *p* < 0.05. Pairwise Mann–Whitney *U*-test **statistically significant difference inter-group at *p* < 0.05

**Fig. 3:**
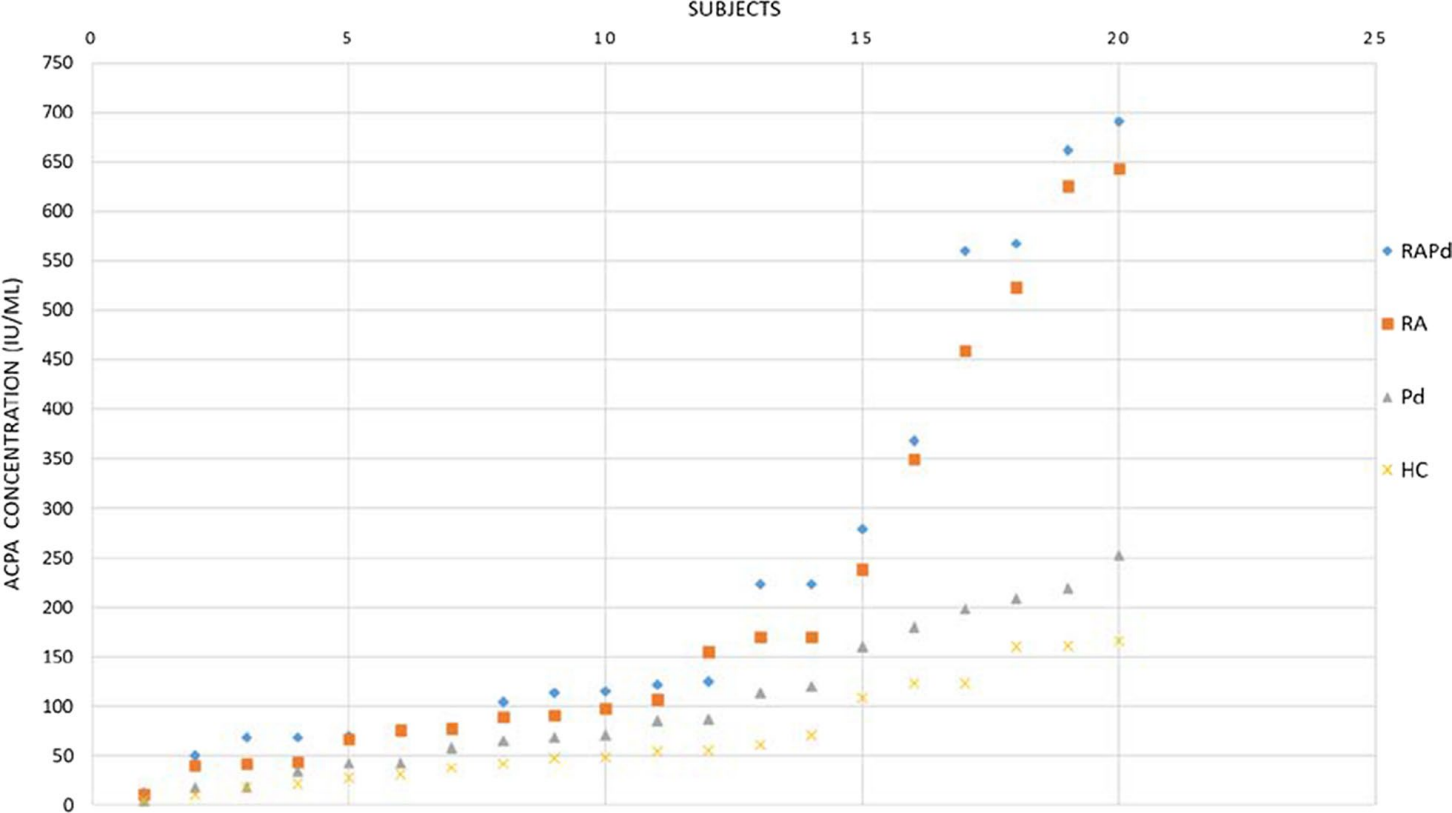
Distribution of serum ACPA concentration in four groups of subjects (in ascending order). There is an increase in the serum ACPA concentration in which the RAPd group had the highest concentration followed by RA group, Pd group and healthy control group (RAPd > RA > Pd > control)

The serum ACPA levels for all four groups are shown in a boxplot (Fig. 2). Median (IQR) for RAPd group was 118.58(274.51) IU/mL, RA group was 102.02(252.89) IU/mL, Pd group was 78.48(132.6) IU/mL, and HC group was 51.67(91.31) IU/mL. Serum ACPA level was significantly higher in RAPd, and RA group compared to that of HC. There is an increase in ACPA levels from HC group, Pd group, RA group to RAPd group. At individual level, the amount of serum ACPA seem to have an increasing trend with the diseased condition in the order of RAPd > RA > Pd > HC (Fig. 3).

### Correlation analysis between ACPA and clinical disease parameters

Bivariate correlation analyses were performed to assess the relationship between serum ACPA levels and recorded clinical parameters of RA (Table 3) and Pd (Table 4). In both RAPd and RA groups, there were no correlations between the serum ACPA levels with ESR and RA disease duration (r < 0.30; *p* > 0.05). Similarly, this present study also demonstrated no significant correlations between serum levels of ACPA with all the clinical periodontal parameters (VPI, GBI, PPD, CAL and PISA) in all the four groups of participants (r < 0.30; *p* > 0.05). Multiple regression analyses were further performed to assess these correlations after adjusting for confounding factors (age, gender, ethnicity, and smoking status) (Additional file 1: Tables S1 and S2). However, these confounding factors did not contribute significantly to the model and were not potential predictors for the variability in serum ACPA, RA parameters and Pd clinical parameters.

**Table 3:** Bivariate correlation analyses of serum anti-citrullinated protein antibodies with clinical RA Parameters

RAPd (n = 20)			RA (n = 20)				
ACPA	r	*p*-value	ACPA	r			*p*-value
ESR	0.242	0.141	ESR	− 0.133			0.577
Disease duration	0.262	0.264	Disease duration	− 0.012			0.961

Spearman rho correlation test; r-value: 0.5–1.0 (strong correlation); − 0.3 to 0.5 (moderate correlation); below 0.3 (weak to no correlation)

RA, Rheumatoid arthritis; Pd, Periodontitis; RAPd, Subjects with RA and Pd; RA, Subjects with RA but without Pd; ACPA, anti-citrullinated protein antibodies; ESR, Erythrocyte sedimentation rate

**Significant at *p* < 0.05

**Table 4:** Bivariate correlation analyses of serum anti-citrullinated protein antibodies with clinical periodontal parameters

	RAPd (n = 20)		RA (n = 20)		Pd (n = 20)		HC (n = 20)	
ACPA	r	*p*-value	r	*p*-value	r	*p-*value	r	*p-*value
VPI	0.228	0.334	0.254	0.28	0.119	0.618	0.085	0.723
GBI	0.052	0.828	− 0.030	0.90	0.045	0.850	− 0.058	0.808
PPD	− 0.03	0.899	− 0.057	0.811	0.051	0.830	− 0.182	0.441
CAL PISA	0.099 0.211	0.678 0.373	0.275 0.137	0.240 0.565	0.107 − 0.032	0.654 0.895	− 0.201 − 0.093	0.196 0.698

Spearman rho correlation test; r-value: 0.5–1.0 (strong correlation); − 0.3 to 0.5 (moderate correlation); below 0.3 (weak to no correlation)

RA, Rheumatoid arthritis; Pd, Periodontitis; RAPd, Subjects with RA and Pd; RA, Subjects with RA but without Pd; Pd, Subjects without RA but has Pd; HC, Subjects without both RA and Pd; ACPA, anti-citrullinated protein antibodies; VPI, visible plaque index; GBI, gingival bleeding index; PPD, probing pocket depth; CAL, clinical attachment level

**Significant at *p* < 0.05

## Discussion

In this cross-sectional comparative study, serum ACPA levels were compared between patients who were diagnosed with Pd, RA, and RAPd. The levels of serum ACPA, from higher to lower, were recorded as follows: RAPd > RA > Pd > HC group. The levels of serum ACPA were found to be statistically significantly higher in the RA cohorts (both RAPd and RA groups) as compared to the HC group. Importantly, high serum ACPA levels in those diagnosed with RA have been demonstrated in other studies [19, 26], It is an established fact that ACPA are present in early RA disease and are highly specific for RA [27]. Biologically, ACPA have been shown to be involved in the inflammatory processes that occur in RA [28, 29]. This finding also reaffirms ACPA as a specific serological marker in RA pathogenesis [12]. Furthermore, earlier it was reported that ACPA may be detectable at low levels in about 1–3% of healthy individuals without any joint symptoms [30, 31].

It has been proposed that periodontal pathogenesis contributes to systemic inflammation by generating citrullinated proteins in inflamed periodontal tissues, subsequently leading to the formation of ACPA [6, 32]. Over the years, researchers have attempted to find possible associations between RA and Pd in relation to ACPA (a common serological marker for RA) and clinical parameters of periodontitis. However, the current study did not show any statistically significant correlations between serum ACPA levels and common clinical Pd parameters namely, VPI, GBI, PPD, CAL and PISA. Similar findings were also reported in earlier studies [33, 34]. It is to be noted that serum ACPA levels of Pd and HC groups are not significantly different. Hence, it is not unlikely that serum ACPA level does not correlate with periodontal clinical parameters. This is contrary to the other studies where a statistically significant correlation between serum ACPA and Pd parameters [16, 30] were shown.

Taken together, it can be concluded that serum ACPA levels are more influenced by RA pathogenesis than that of Pd. Notably, generation of ACPA in RA and Pd is expected to be different. This is based on the notion that, ACPA is the product of host mediated citrullination in RA, while the same has been suggested to be a product of citrullination mediated by the periodontal pathogens in periodontitis.

In view of the plausibility of Pd as a risk factor that causes systemic inflammatory responses and cross reactivity in RA [35, 36], PISA was calculated to quantify the amount of inflamed periodontal tissue and as such, quantifies the systemic inflammatory burden. Following this biological model, the larger the amount of inflamed periodontal tissue there is, the higher the chances of Pd eliciting systemic inflammatory responses and autoimmune responses [37] that may contribute to the increased ACPA levels. Once again, the current study did not find any statistically significant correlations between serum levels of ACPA with PISA in all four groups. This could be due to the recruitment of gingivitis cases in our healthy control group which has contributed to a higher PISA score in HC group than the RA group. Furthermore, PISA might not be the sole determinant in predicting the plausibility of Pd as a risk factor for systemic disease even though it can measure the amount of inflamed periodontal tissues. For instance, the type of inflammation might be more crucial than the amount of inflammation as PISA does not consider the types of microflora, cells, proteins and inflammatory mediators that might play a key role in causing inflammation [24, 38]. Our study groups of Pd and RA were also heterogenous as they come from different ethnic groups.

Like earlier reports, the current study demonstrated that serum ACPA and ESR (a common clinical parameter for RA) of RAPd and RA groups are not corelated [39, 40]. This finding reaffirms the notion that the quality of the ACPA response defined by molecular characteristics or functional features of ACPA expressing autoreactive B cells are much more important and relevant than its quantity (serum level of ACPA) in determining the outcome of established RA. It has been shown that the fluctuations in serum ACPA levels do not reflect RA disease activity and are not clinically useful in predicting the progression and flare-up of the disease [41]. Therefore, measurements of serum ACPA may not be useful in monitoring RA disease activity. This warrants more research to develop more inflammatory markers for RA.

Given the sample number and contradictory observations with the existing literature, it might not be prudent to extrapolate the current observation at a population level. While the current study warrants further investigation with larger samples to resolve whether the contradiction is solely due to the limited sample size. Future study that using a larger sample size from multiple centres may provide better validation of the findings. Moreover, inclusion of additional data on the inflammatory markers such as DAS-28 and CRP could have been more useful to analyse the severity of RA pathogenesis. Although ESR is a good inflammatory marker that can reflect RA disease activity over the preceding weeks, it could be affected by other confounding factors such as age, gender, fibrinogen levels and rheumatoid factor (RF) [42]. Lastly, none of the case definitions/classifications that are being used currently suffice to define the degree of inflamed periodontal tissue and hence may not be able to quantify the inflammatory burden posed by periodontitis. Thus, it is proposed that PISA can be used in conjunction with the case definition/classification when Pd is investigated as a potential risk factor for RA or any other systemic diseases.

## Conclusion

Lack of correlation between ACPA with the clinical parameters of Pd suggests that citrullination of proteins during Pd pathogenesis might not be sufficient to make a mechanistic or causal link between RA and Pd. Further studies using more sensitive biomarkers that are common in both clinical conditions are needed to establish the role of Pd in the development and progression of RA.

## Supplementary Information

**Additional file 1: Table S1**. Multiple linear regression analysis models for ACPA, ESR and disease duration controlling for age, gender, ethnicity, and smoking status in the RAPd and RA groups. These confounding factors did not contribute significantly to the model. RAPd: Subjects with RA and Pd; RA: Subjects with RA but without Pd; ACPA: anticitrullinated protein antibodies; ESR: Erythrocyte sedimentation rate; Multiple linear regression analysis, **significant at* p* < 0.05; B = Unstandardised regression coefficients; β= Standardised regression coefficients; CI = Confidence interval.** Table S2**. Multiple regression analysis models for periodontal parameters controlling for age, gender, ethnicity, and smoking status in all groups (RAPd, RA, Pd and HC). All these confounding factors did not contribute significantly to the model and were not potential predictors for the variability in clinical periodontal parameters. RAPd: Subjects with RA and Pd; RA: Subjects with RA but without Pd; Pd: Subjects without RA but has Pd; HC: Subjects without both RA and Pd; VPI: visible plaque index; GBI: gingival bleeding index; PPD: probing pocket depth; CAL: clinical attachment level; PISA: Periodontal inflamed surface area; Multiple linear regression analysis, **significant at* p* < 0.05; B = Unstandardised regression coefficients; β= Standardised regression coefficients; CI = Confidence interval.

## Data Availability

The datasets generated during and analyzed during the current study are not publicly available due to containing information that could compromise the privacy of research participants but are available from the corresponding author on reasonable request.

## Abbreviations

ACPA: Anti-citrullinated protein antibodies
ACR/EULAR: American College of Rheumatology/European League Against Rheumatism
BOP: Bleeding on probing
CAL: Clinical attachment loss
CI: Confidence interval
CRP: C-reactive protein
DAS: Disease activity score
DMARDs: Disease modifying antirheumatic drugs
ELISA: Enzyme-linked immunosorbent assay
ESR: Erythrocyte sedimentation rate
GBI: Gingival Bleeding Index
HC: Subjects without RA and Pd/healthy individuals
IQR: Interquartile range
MREC: Medical Research Ethics Committee
NSAIDs: Non-steroidal anti-inflammatory drugs
OD: Optical density
PADs: Peptidyl arginine deiminase enzymes
Pd: Periodontitis
PISA: Periodontal inflamed surface area
PPD: Probing pocket depth
RA: Rheumatoid arthritis
RAPd: Participants with RA and Pd
RF: Rheumatoid factor
SD: Standard deviation
SPSS: Statistical Package of Social Sciences
UMMC: University of Malaya Medical Centre
VPI: Visible Plaque Index
