# Supplementary material for: Antibodies against citrullinated proteins in relation to periodontitis with or without rheumatoid arthritis: a cross-sectional study
Source: BMC Oral Health. 2021 Jul 20;21:360. doi: 10.1186/s12903-021-01712-y (PMC8293567; doi:10.1186/s12903-021-01712-y)
Supplement: Supplementary file 1 — Additional file 1: Table S1. Multiple linear regression analysis models for ACPA, ESR and disease duration controlling for age, gender, ethnicity, and smoking status in the RAPd and RA groups. These confounding factors did not contribute significantly to the model. RAPd: Subjects with RA and Pd; RA: Subjects with RA but without Pd; ACPA: anticitrullinated protein antibodies; ESR: Erythrocyte sedimentation rate; Multiple linear regression analysis, **significant at p < 0.05; B = Unstandardised regression coefficients; β= Standardised regression coefficients; CI = Confidence interval. Table S2. Multiple regression analysis models for periodontal parameters controlling for age, gender, ethnicity, and smoking status in all groups (RAPd, RA, Pd and HC). All these confounding factors did not contribute significantly to the model and were not potential predictors for the variability in clinical periodontal parameters. RAPd: Subjects with RA and Pd; RA: Subjects with RA but without Pd; Pd: Subjects without RA but has Pd; HC: Subjects without both RA and Pd; VPI: visible plaque index; GBI: gingival bleeding index; PPD: probing pocket depth; CAL: clinical attachment level; PISA: Periodontal inflamed surface area; Multiple linear regression analysis, **significant at p < 0.05; B = Unstandardised regression coefficients; β= Standardised regression coefficients; CI = Confidence interval. [file 12903_2021_1712_MOESM1_ESM.docx]

**Additional file 1: Table S1:** **Multiple regression analysis models for ACPA, ESR and disease duration controlling for age, gender, ethnicity and smoking status in the RAPd and RA groups (N=40)**

| Variables | *B* [95% CI] | β | t | *p*-value |
| --- | --- | --- | --- | --- |
| 1. **ACPA** |  |  |  |  |
| Age | 1.71[-1.49,4.91] | 0.12 | 0.996 | 0.290 |
| Gender | -28.18[-95.55,77.47] | 0.023 | -0.574 | 0.836 |
| Ethnicity | -2.845[-57.16, 51.46] | 0.013 | -0.104 | 0.917 |
| Smoking status | 19.28[-27.67,66.22] | 0.105 | 0.811 | 0.416 |
| 1. **ESR** |  |  |  |  |
| Age | 0.083[-.572,.737] | 0.041 | 1.931 | 0.799 |
| Gender | -13.89[-30.97,3.186] | -0.312 | 0.257 | 0.108 |
| Ethnicity | -7.077 [-14.534, | 0.38 | -0.310 | -1.651 |
| Smoking status | 4.377[-5.194,13.947] | 0.175 | -1.927 | 0.360 |
| 1. **Disease duration** |  |  |  |  |
| Age | 0.022[-.009,.053] | 0.235 | 1.446 | 0.157 |
| Gender | 0.079[-0.724,0.882] | 0.038 | 0.200 | 0.843 |
| Ethnicity | 0.020[-0.330,0.370] | 0.019 | 0.115 | 0.909 |
| Smoking status | -0.271[-0.721,0.179] | -0.235 | -1.223 | 0.230 |

Multiple linear regression analysis, **significant at *p*<0.05; B = Unstandardised

regression coefficients; β= Standardised regression coefficients; CI= Confidence interval.

**Additional file 1: Table S2: Multiple regression analysis models for periodontal parameters controlling for age, gender, ethnicity, and smoking status in all groups (RAPd, RA, Pd and HC) (N=80)**

| Variables | *B* [95% CI] | β | t | *p*-value |
| --- | --- | --- | --- | --- |
| 1. **VPI** |  |  |  |  |
| Age | 0.192[-0.349,0.734] | 0.085 | 0.708 | 0.481 |
| Gender | -11.179[-26.92,44.56] | -0.184 | -1.414 | 0.161 |
| Ethnicity | -2.322[-10.875,6.230] | -0.065 | -0.541 | 0.590 |
| Smoking status | 2.307[-5.137,9.750] | 0.080 | 0.617 | 0.539 |
| 1. **GBI** |  |  |  |  |
| Age | -0.207[-0.675,0.261] | -0.105 | -0.880 | 0.382 |
| Gender | -9.365[-22.995,4.265] | -0.177 | -1.369 | 0.175 |
| Ethnicity | 0.554[-6.850,7.958] | 0.018 | 0.149 | 0.882 |
| Smoking status | -0.485[-6.928,5.959] | -0.019 | -0.150 | 0.881 |
| 1. **Mean PPD** |  |  |  |  |
| Age | -0.004[-0.023,0.015] | -.047 | -0.407 | 0.685 |
| Gender | -0.686[-1.237, 0.135] | -.313 | -2.480 | 0.515 |
| Ethnicity | -0.035[-0.335,0.264] | -0.028 | -0.235 | 0.815 |
| Smoking status | 0.036[-0.225,0.296] | 0.034 | 0.275 | 0.784 |
| 1. **Mean CAL** |  |  |  |  |
| Age | 0.027[-0.013,0.067] | 0.156 | 1.342 | 0.184 |
| Gender | -1.225[-2.388, 0.062] | -0.264 | -2.098 | 0.069 |
| Ethnicity | -.033[-0.665,0.598] | -0.012 | -0.105 | 0.916 |
| Smoking status | -.012[-0.562,0.538] | -0.006 | -0.045 | 0.964 |
| 1. **PISA** |  |  |  |  |
| Age | -8.903[-22.294,4.488] | -0.157 | -1.324 | 0.189 |
| Gender | 297.99[687.612,91.631] | -0.195 | -1.524 | 0.132 |
| Ethnicity | 40.101[171.538,251.739] | 0.045 | 0.377 | 0.707 |
| Smoking status | 8.777[-175.414,192.969] | 0.012 | 0.095 | 0.925 |

Multiple linear regression analysis, **significant at *p*<0.05; B = Unstandardised

regression coefficients; β= Standardised regression coefficients; CI= Confidence interval.
